# A regionally-adapted implementation of conservation agriculture delivers rapid improvements to soil properties associated with crop yield stability

**DOI:** 10.1038/s41598-018-26896-2

**Published:** 2018-05-31

**Authors:** Alwyn Williams, Nicholas R. Jordan, Richard G. Smith, Mitchell C. Hunter, Melanie Kammerer, Daniel A. Kane, Roger T. Koide, Adam S. Davis

**Affiliations:** 1 Department of Agronomy and Plant Genetics, University of Minnesota, St Paul, MN USA; 2 School of Agriculture and Food Sciences, The University of Queensland, Gatton, QLD Australia; 3 Department of Natural Resources and the Environment, University of New Hampshire, Durham, NH USA; 4 Department of Plant Science, The Pennsylvania State University, University Park, PA USA; 5 Department of Plant, Soil and Microbial Sciences, Michigan State University, East Lansing, MI USA; 6 Department of Biology, Brigham Young University, Provo, UT USA; 7 USDA-ARS, Global Change and Photosynthesis Research Unit, Urbana, IL USA

## Abstract

Climate models predict increasing weather variability, with negative consequences for crop production. Conservation agriculture (CA) may enhance climate resilience by generating certain soil improvements. However, the rate at which these improvements accrue is unclear, and some evidence suggests CA can lower yields relative to conventional systems unless all three CA elements are implemented: reduced tillage, sustained soil cover, and crop rotational diversity. These cost-benefit issues are important considerations for potential adopters of CA. Given that CA can be implemented across a wide variety of regions and cropping systems, more detailed and mechanistic understanding is required on whether and how regionally-adapted CA can improve soil properties while minimizing potential negative crop yield impacts. Across four US states, we assessed short-term impacts of regionally-adapted CA systems on soil properties and explored linkages with maize and soybean yield stability. Structural equation modeling revealed increases in soil organic matter generated by cover cropping increased soil cation exchange capacity, which improved soybean yield stability. Cover cropping also enhanced maize minimum yield potential. Our results demonstrate individual CA elements can deliver rapid improvements in soil properties associated with crop yield stability, suggesting that regionally-adapted CA may play an important role in developing high-yielding, climate-resilient agricultural systems.

## Introduction

Climate change models predict increasing intra- and inter-annual weather variability in the coming decades, with negative consequences for global crop production^1^. Such variability includes greater incidence of heat stress, periods of drought and severe rainfall events^2–4^. Maintaining high levels of agricultural productivity under conditions of variable weather (i.e., crop yield stability) is critical for meeting increasing global demand for agricultural products, including food, fuel and fiber^5^. While intensive public and private programs have made significant improvements to crop germplasm to help adapt agriculture to climate change^6,7^, much less progress has been made on climate-resilient agroecosystem management.

Conservation agriculture (CA) is a broadly applicable approach to agroecosystem management that may improve climate resilience^8^. Conservation agriculture can potentially promote climate resilience by increasing rainfall infiltration and soil moisture holding capacity, reducing anoxia and other hazards related to excessive soil moisture, moderating soil temperature fluctuations, and improving soil nutrient cycling processes^9^. Many of these resilience-promoting effects may result from CA’s impacts on soil structure and soil organic matter (SOM) concentration. Indeed, recent research has revealed that SOM is important to crop yield stability^10,11^. Thus, increases in SOM should enable a given soil to sustain higher and more stable levels of crop production under adverse environmental conditions than the same soil with less SOM.

SOM can be increased by the three key elements that constitute CA as it is currently understood. These elements are limiting tillage intensity, maintaining soil cover with crop residues or cover crops, and increasing agroecosystem crop diversity via crop rotation^12^. No- and reduced tillage practices such as zonal tillage^13^ significantly reduce soil disturbance compared with more intense practices such as moldboard and chisel plough, allowing SOM accumulation or reduced SOM depletion in surface soil layers^14,15^. Reduced tillage intensity also improves soil structure, increasing the capacity of soil to both drain effectively and retain moisture^16^. Cover cropping extends the continuity of living plant cover on fields that would otherwise be fallow over winter, providing additional inputs of fresh organic matter that can help build SOM stocks^17,18^. Lastly, implementing crop rotations can improve soil structure, build SOM and enhance indicators of soil fertility^17,18^.

The key elements of CA, while having potential to improve SOM, may also impose yield costs when applied individually. For example, yield costs have been associated with complete avoidance of soil disturbance^19^, and with implementation of cover cropping^20^. However, emerging evidence suggests that fully-implemented CA—including all three key elements—will produce yields similar to those in conventional, non-CA cropping systems^19^.

Several recent studies have found that fully-implemented CA can improve crop yield stability—a measure of climate resilience—in certain soil types, climates and cropping systems^21–24^. These findings suggest that fully-implemented CA might promote climate resilience without yield cost, but deeper analysis is needed to guide management and further refine regionally-adapted CA. Specifically, more evidence is needed of whether, and how quickly, CA can lead to improvements in soil properties that enhance climate resilience. While relationships between soil properties and yield stability have been discussed in the past^25–27^, few studies have evaluated causal linkages between individual CA elements, changes in soil properties, and subsequent impacts on crop yield stability^11,21^. Given the costs and risks associated with changing management practices, the absence of tangible improvements in the short-term (<5 years) may limit grower adoption of CA^28,29^. Moreover, the large diversity of cropping systems in which CA is implemented makes is hard to draw regionally-relevant conclusions^30^.

To address these knowledge gaps, we examined a regionally-adapted CA system, using a robust experimental design to explore causal linkages between individual CA elements, changes in soil properties, and subsequent impacts on crop yield stability. Specifically, across four states in the eastern and Midwestern US, we investigated the short-term (<5 years) impacts of four different management systems with differing levels of CA implementation on soil properties associated with crop yield stability. We also investigated soybean (*Glycine max* L. Merr.) and maize (*Zea mays* L.) yields and indicators of yield stability. Our management systems ranged from conventional (chisel plough with no cover crops) to regionally-adapted CA (reduced tillage with cover crops). Our regionally-adapted CA system involved an integration of zonal soil management^13^ (limited and targeted soil disturbance) and cover cropping. This integration reduces tillage intensity relative to chisel plough, thereby enhancing soil building processes, and overcomes some of the downsides of no-tillage while maintaining soil cover with crop residues and cover crops^13,31^. All systems utilized a maize—soybean crop rotation, which is the dominant crop rotation in the eastern and Midwestern US. Thus, while our experiment did not assess the crop rotation element of CA, it did assess the tillage and cover crop elements within an economically-viable crop rotation in our study region; hence, our experiment assessed a regionally-adapted implementation of CA. The US is one of the world’s most important crop production regions, accounting for approximately 35% of global maize and soybean production in 2014^32^.

We hypothesized that, during our five-year experiment, (1) Cover cropping and reduced tillage would improve soil hydrothermal properties (plant-available water and temperature) and fertility (cation exchange capacity (CEC), nutrient availability, and pH); (2) These soil changes would increase crop yield stability in the face of water- and nutrient-stress; and (3) Cover cropping and reduced tillage would not adversely affect crop yields.

## Materials and Methods

### Field sites and experimental design

Experimental plots were established in 2012 at four sites across the eastern and Midwestern US: Savoy (Illinois), Mason (Michigan), Rosemount (Minnesota) and Rock Springs (Pennsylvania), providing wide variation in soil types and climate; soil taxonomic and climate data for each site are provided in Table 1. At each site, two tillage and two cover crop treatments were established in a complete randomized block design, with four replicate blocks per site. The two tillage treatments were chisel plough and ridge tillage, as examples of conventional and reduced tillage, respectively. Cover crop treatments consisted of no cover crop (winter fallow) or winter cereal rye (*Secale cereale* L.). Within each block were a total of eight plots: four planted to maize and four planted to soybean, with crops rotated annually. This gave a total of 4 × 8 = 32 plots at each site. During the maize phase of the rotation each plot received inorganic nitrogen (N) fertiliser; during the soybean phase of the rotation, plots received no N fertiliser. Management at each site followed local best management practices. Detailed plot-level information, including N fertiliser amounts and equipment used, are presented in Williams *et al*.^33^.

**Table 1:** Soil taxonomic and climate data for the four sites and coordinates of their locations.

Site	Soil series	Soil type	Precip. (cm)	Temp. (°C)	Location
Illinois	Drummer	Silty clay loam	61.6	18.3	40° 3′, −88° 15′
Michigan	Marlette	Sandy loam	48.0	17.3	42° 24′, −85° 24′
Minnesota	Waukegan	Silty clay loam	69.0	16.9	44° 44′, −93° 7′
Pennsylvania	Hagerstown	Coarse silt loam	55.0	17.9	40° 47′, −77° 51′

Precipitation and temperature figures are the 30-year means for the growing season (April-October in IL; May-October for MI, MN and PA).

### Soil sampling and analysis

Soil samples (0–10 cm depth) were collected at all four sites in October 2011, prior to the initiation of our experimental treatments, and again in October 2015. Samples were sieved to 2.5 mm and air-dried before being sent to Waypoint Analytical (Memphis, Tennessee, USA) for analysis of SOM (loss on ignition), pH (1:1 w/v H_2_O), phosphorus (P; Mehlich 3 extraction), and cation exchange capacity (CEC). The analysis of soil samples in 2011 and 2015 allowed us to investigate changes in SOM due to four years of our experimental treatments. We analysed data from 0–10 cm depth because this layer has been shown to be highly responsive to agronomic management^15^ and also contains a high density of crop roots^34^.

Soil moisture (0–10 cm depth) in maize plots was measured using volumetric soil moisture sensors (Decagon ECH2O™, S-SMC-M005, Onset Computer Corporation, Bourne, MA, USA; two sensors per plot, in a crop row and inter-row). Readings were taken every minute and integrated hourly using a miniature data logger (HOBO micro-station logger; #H21-002; Onset Computer Corporation, Bourne, Massachusetts, USA). Hourly readings were aggregated to calculate daily means. Soil temperatures (0–10 cm depth) were also measured continuously in maize plots (HOBO Pendant Logger; UA-001-64), with hourly readings aggregated to generate daily means. Mean daily soil moisture and temperature values for each maize plot were then calculated across years for the period between the maize six leaf stage (V6) and tasseling (VT). This growing season period encompasses the period of peak maize N demand and biomass accumulation^35^.

### Crop yields and yield stability

Maize was harvested at full physiological grain maturity, designated by the development of a black abscission layer at the base of kernels. Maize ears were hand harvested within two 3 m long rows. Kernels were mechanically separated from cobs, and fresh grain mass determined. Grain was then dried to constant mass in a forced air oven to determine dry mass. Maize yields were expressed in kg ha^−1^ at 15.5% moisture content. Fully senesced soybean plants were hand harvested within two 3 m long rows, and processed with a stationary thresher to remove grain. Grain fresh and dry mass for soybean were determined in the same way as for maize, and soybean yields were expressed in kg ha^−1^ at 13% moisture content.

Minimum yield potential and temporal yield variability were used as indicators of crop yield stability, and were calculated for each tillage and cover crop treatment combination following Williams *et al*.^11^. In summary, adaptability analysis was used to quantify minimum yield potential (crop yields under the poorest growing conditions over the period of the experiment), where for each site annual maize or soybean yield averages were used to create an environmental index ranking production years from ‘poor’ to ‘good’^36^. Crop yield responses to the environmental index were then developed for each tillage-cover crop treatment combination using linear mixed effects models with random intercepts and slopes. Model predictions at the minimum end of the environmental index were extracted and used as our measure of minimum yield potential. Temporal yield variability was quantified for maize and soybean as the coefficient of variation (yield standard deviation/mean yield) for each tillage-cover crop treatment combination over the duration of the study.

### Statistical analysis

Our analysis followed a three-step process: (1) identify changes in soil properties associated with crop yield stability due to the experimental treatments; (2) determine crop yield stability and crop mean yield responses to the experimental treatments; (3) investigate relationships between yield stability and soil properties to elucidate pathways through which CA may influence climate-resilient yields.

Changes in SOM from 2011 to 2015 were assessed using delta values (i.e., Δ SOM = value of SOM in 2015 – value of SOM in 2011; thus, a positive Δ indicates that SOM increased from 2011 to 2015, whereas a negative Δ indicates that SOM decreased from 2011 to 2015). Δ SOM, in addition to mean 2015 values for CEC, pH and P, as well as soil moisture and temperature over the V6 and VT period (means over 2012–2015), were assessed against tillage and cover crop treatments using linear mixed effects models fitted with restricted maximum likelihood estimations and site as a random effect (α < 0.05). An interaction term was fitted to the fixed effects, allowing analysis of the individual and combined effects of tillage and cover crop.

Maize and soybean minimum yield potential and temporal yield variability over the 2012–2015 period were each assessed against the experimental treatments using linear mixed effects models (fitted as described above for soil properties). For maize and soybean yields, we analysed the full data set of annual means by tillage and cover crop treatments. Yields were analysed with linear mixed effects models fitted with restricted maximum likelihood estimations, using a nested year/site/block random effects structure.

**Figure 1:**
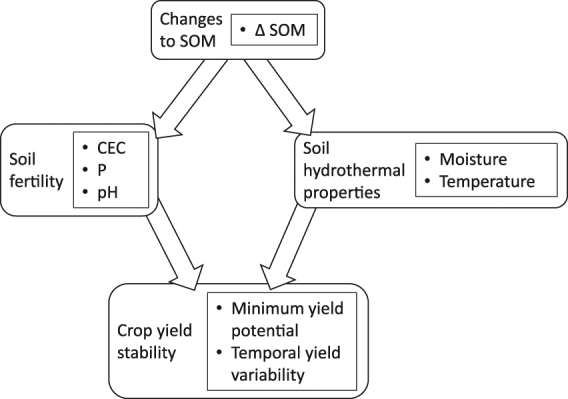
Conceptual model representing how changes in soil organic matter from 2011 to 2015 (Δ SOM) can affect crop yield stability via soil fertility and soil hydrothermal properties. CEC: cation exchange capacity; P: soil phosphorus.

Associations between soil properties that showed significant changes due to the experimental treatments and our metrics of yield stability were assessed using structural equation modeling^37^. Our analyses were guided by a conceptual model of causal links between soil properties and yield stability, depicted in Fig. 1. The model was predicated on the notion that changes in SOM concentrations influences yield stability via two separate pathways: soil fertility (CEC, pH and P) and soil hydrothermal (plant-available water and temperature) conditions. These pathways would increase crop yield stability in the face of nutrient- and water-stress, respectively. By using a structural equation modeling approach, we could identify the relative importance of each of these pathways^37^. Data from all four sites were pooled to increase our effective sample size. This was achieved by extracting the residuals of one-way ANOVAs where site was fitted as the explanatory variable, thereby partialling out the variation in the data attributable to site. The pooled (site-partialled) data was then used for structural equation modeling. Models were simplified using maximum likelihood and Akaike (AIC) weights until the most parsimonious model was found. Separate models were fitted for maize and soybean. All analyses were conducted in R 3.3.1^38^, using the lavaan^39^ and nlme^40^ packages.

The datasets generated and analysed during the current study are available in the USDA National Agricultural Library AgDataCommons repository: https://data.nal.usda.gov/dataset/regionally-adapted-implementation-conservation-agriculture-delivers-rapid-improvements-soil-properties-associated-crop-yield-stability.

## Results

### Effects of CA practices on soil properties

**Figure 2:**
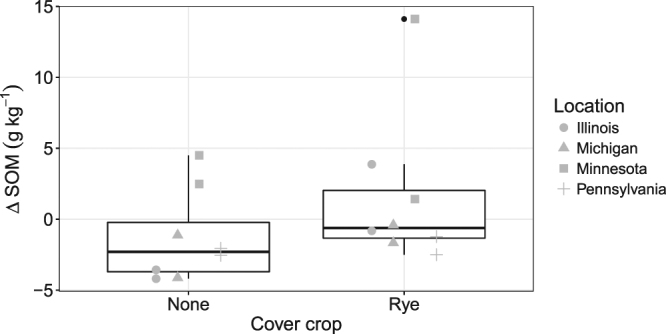
Changes in soil organic matter (Δ SOM, 0–10 cm depth) from 2011 to 2015 by cover crop treatment.

Tillage had no effect on SOM levels between 2011 and 2015. However, cover cropping increased SOM relative to no cover crop (*t*_1,9_ = 3.12, *P* = 0.012), resulting from either SOM accretion or a reduced rate of SOM depletion (Fig. 2). CEC in 2015 was marginally greater in the cereal rye cover crop treatment compared with the no cover crop treatment (*t*_1,11_ = 2.37, *P* = 0.037; Fig. S1); CEC was unaffected by tillage. Soil pH and P in 2015 were unaffected by tillage or cover cropping.

Mean soil moisture and temperature over the V6 to VT period differed by treatment. Mean daily soil moisture was greater under ridge tillage than chisel plough (*t*_1,11_ = 3.35, *P* = 0.007; Fig. S2), while mean daily soil temperature was marginally lower in plots that received a cereal rye cover crop (*t*_1,11_ = 2.24, *P* = 0.047; Fig. S3).

### Yield stability and crop yields

**Figure 3:**
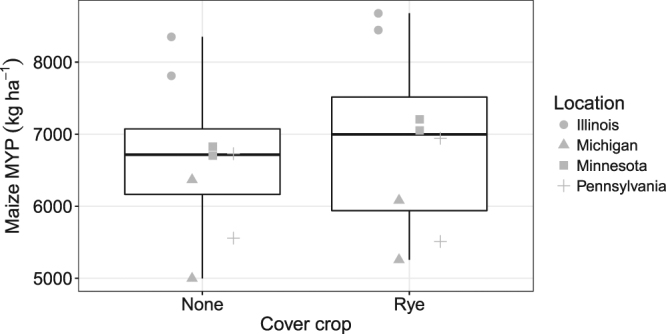
Maize minimum yield potential (MYP) over 2012–2015 by cover crop treatment.

**Figure 4:**
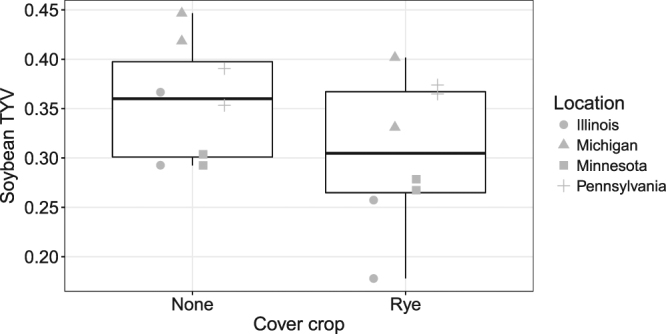
Soybean temporal yield variability (TYV) over 2012–2015 cover crop treatment.

Yield stability was estimated along two dimensions: minimum yield potential and temporal yield variability. For maize, minimum yield potential was unaffected by tillage but was greatest in plots that received a cereal rye cover crop (*t*_1,11_ = 4.15, *P* = 0.002, Fig. 3). Maize temporal yield variability was unaffected by tillage or cover cropping. For soybean, minimum yield potential was unaffected by tillage or cover cropping, while temporal yield variability was significantly lower in cereal rye cover cropped plots (*t*_1,11_ = 3.54, *P* = 0.005; Fig. 4).

Mean maize yields did not differ by cover cropping but were marginally lower under ridge tillage compared with chisel plough (*t*_1,215_ = 2.00, *P* = 0.047; Fig. S4). For soybean, mean yields were unaffected by tillage or cover crop treatments (Fig. S5).

### Relationships between soil properties and crop yield stability

Guided by our conceptual model of causal links between soil properties and yield stability (Fig. 1), and the response of these variables to our tillage and cover crop treatments, the following soil variables were incorporated into structural equation models: Δ SOM, CEC in 2015, and mean soil moisture and temperature over the V6 to VT period. Via structural-equation modeling, the explanatory power of these variables was explored by using them to explain observed differences in maize minimum yield potential and soybean temporal yield variability as a result of management treatments with or without reduced tillage and cover cropping (Fig. 1).

**Figure 5:**
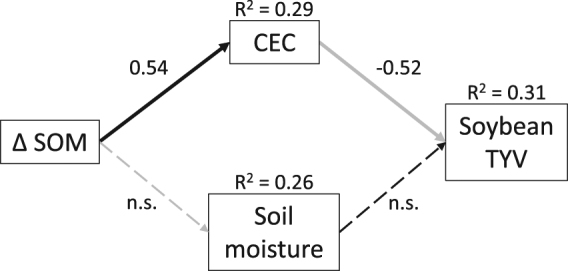
Structural equation model showing relationship between changes in soil organic matter from 2011 to 2015 (Δ SOM) and soybean temporal yield variability (TYV) via associations with soil cation exchange capacity (CEC) and soil moisture. Black arrows indicate positive relationships; gray arrows indicate negative relationships. Solid arrows indicate significant (*P* < 0.05) relationships; broken arrows indicate non-significant (n.s.) relationships. Numbers along arrows show standardized coefficients.

For soybean, structural equation modeling revealed that changes in SOM due to cereal rye cover cropping were associated with a reduction in temporal yield variability via increases in CEC (Fig. 5). Soil temperature was removed from the starting model for parsimony, and while soil moisture was retained in the model, it had no significant relationships with Δ SOM or temporal yield variability (Fig. 5). Soil P and pH were not included in the starting model as they showed no response to experimental treatments (see above). The soybean model was well supported by the data (χ^2^ = 2.66, df = 2, *P* = 0.27). For maize, in contrast, SEM did not indicate any relationships between the soil variables and maize minimum yield potential.

## Discussion

In this study, we identified clear effects of the tillage and cover crop elements of CA on soil properties associated with crop yield stability. Further, we also identified linkages between those effects and crop yield stability. These effects were consistent across four US states, spanning a globally significant production region with wide variation in soil and climate conditions. Maize and soybean responded differently to the tillage and cover crop treatments, indicating that approaches to successfully implementing CA may be crop dependent.

With respect to our first hypothesis, that cover cropping and reduced tillage would improve soil hydrothermal properties and fertility, we found strong support. Compared with chisel plough, ridge tillage led to marked improvement in the capacity of soils to store water for crop growth. Such a result was previously demonstrated at the same experimental sites after only two years of implementing ridge tillage^33^; our current result demonstrates the durability of that change over four years. Increased storage of soil water may be due to improvements in soil structure resulting from a reduction in tillage-based soil disturbance^41^. Cover cropping with cereal rye led to an increase in SOM relative to non-cover cropped plots, as well as an increase in CEC and reduction in soil temperatures. Cover cropping can enhance SOM formation by increasing inputs of below-ground carbon, both directly via root material and by enhancing microbial turnover^18,42^. Absolute increases in SOM due to cover cropping were observed in Illinois and Minnesota; while in Michigan and Pennsylvania, cover cropping reduced the rate of SOM decline. However, the combination of reduced tillage and cover cropping did not result in changes to soil properties beyond those induced by either reduced tillage or cover cropping alone. Thus, in this case, the individual elements of CA were more important than their combined implementation.

For our second hypothesis, that changes in soil properties resulting from reduced tillage and cover cropping would increase crop yield stability, we also found strong support. The integration of winter cereal rye as a cover crop, regardless of tillage system, was related to a reduction in soybean temporal yield variability and an increase in maize minimum yield potential. For soybean, this result accords with Gaudin *et al*.^21^, who found that incorporating winter wheat into a maize-soybean rotation increased soybean yield stability. We hypothesized that cover cropping would affect crop yield stability via changes in SOM^10^, which would increase soil moisture retention and fertility. Our results showed partial support for this, with significant improvements in SOM in cover cropped plots compared with non-cover cropped plots across all sites, and regardless of tillage system. However, the influence of SOM on soybean yield stability was related to changes in soil fertility only, with no relationship found between yield stability and soil moisture retention. Reductions in soybean temporal yield variability were related to increases in CEC, and increases in CEC were driven by greater SOM. A recent analysis of numerous long-term (20–30 years) field experiments found that increases in soil fertility via improvements in SOM resulted in greater yield stability of a range of cereal crops^43^. Here we demonstrate that enhancing SOM via cover cropping can produce rapid improvements in soybean yield stability. For maize, cover cropping was found to increase minimum yield potential. However, SEM was unable to identify any relationships between maize minimum yield potential and our measured soil properties. Further analysis of a wider array of soil and maize variables is warranted to identify the mechanism underlying this result.

For our third hypothesis, that cover cropping and reduced tillage would not adversely affect crop yields, we found partial support. Soybean was equally productive across all four management systems. This is an important result, as it indicates that improvements in soybean yield stability did not come at the expense of lower yields. In contrast, under ridge tillage, maize showed a slight yield decline compared with chisel plough, with yields 331 kg ha^−1^ lower across all site-years. Long-term (30 years) maize production data from the US Midwest state of Iowa shows that chisel plough systems typically yield approximately 200 kg ha^−1^ more than ridge tillage systems^44^. Thus, the gap in maize yields seen between our tillage systems may be typical for this production region. Slightly cooler soil temperatures at the time of planting have been observed in ridge tillage systems compared with chisel plough systems at the same experimental sites^33^. While these cooler temperatures did not translate into differences in growing degree days^33^, small reductions in temperature have been shown to reduce maize growth in the first eight weeks after planting^45^. Thus, factors associated with maize emergence and early development may play a more important role in yield determination under conditions where summer hydrothermal and nutritional requirements are not limiting. Indeed, the ridge tillage systems at our sites have been demonstrated to consistently enhance soil N turnover and availability compared with chisel plough, resulting in greater maize tissue N^46,47^. Further exploration of such mechanistic aspects will be required to fully assess the value of CA as a system for soil management and climate resilience.

In summary, we have demonstrated the potential for CA to deliver rapid (<5 years) improvements in soil properties associated with yield stability. Furthermore, we found clear and consistent linkages between changes in soil properties and improvements in soybean yield stability, with no negative effects on soybean productivity. Our results revealed that increases in soybean yield stability were associated with increases in CEC driven by improvements in SOM via cover cropping. The absence of a soybean yield penalty when implementing cover cropping is an important result, as it suggests that conventional farmers may be able to integrate individual elements of CA without sacrificing yields. This would minimize some of the costs of adoption while delivering rapid returns in terms of improved soil properties and soybean yield stability. In contrast, while maize yield stability was improved by cover cropping, we were unable to identify any relationship with changes in soil properties; thus, the mechanistic basis of this effect is unclear. In addition, while cover cropping did not affect maize yields, ridge tillage led to a marginal yield penalty under our experimental conditions, indicating that the impacts of CA are crop specific. Our results suggest that CA may play an important role in the development of high-yielding, climate-resilient agricultural systems, but further research is required to develop crop-specific and regionally-adapted CA approaches.

## Electronic supplementary material

Supplementary Information
