# Supplementary material for: A regionally-adapted implementation of conservation agriculture delivers rapid improvements to soil properties associated with crop yield stability
Source: Sci Rep. 2018 May 31;8:8467. doi: 10.1038/s41598-018-26896-2 (PMC5981580; doi:10.1038/s41598-018-26896-2)
Supplement: Supplementary file 1 — Supplementary Information [file 41598_2018_26896_MOESM1_ESM.pdf]

Supplementary information for:

A regionally-adapted implementation of conservation agriculture delivers rapid improvements to soil properties associated with crop yield stability

Alwyn Williams[1,2]*, Nicholas R. Jordan[1], Richard G. Smith[3], Mitchell C. Hunter[4], Melanie Kammerer[4], Daniel A. Kane[5], Roger T. Koide[6] and Adam S. Davis[7]

[1] Department of Agronomy and Plant Genetics, University of Minnesota, St Paul, MN, USA
[2] School of Agriculture and Food Sciences, The University of Queensland, Gatton, QLD, Australia
[3] Department of Natural Resources and the Environment, University of New Hampshire, Durham, NH, USA
[4] Department of Plant Science, The Pennsylvania State University, University Park, PA, USA
[5] Department of Plant, Soil and Microbial Sciences, Michigan State University, East Lansing, MI, USA
[6] Department of Biology, Brigham Young University, Provo, UT, USA
[7] USDA-ARS, Global Change and Photosynthesis Research Unit, Urbana, IL, USA

***Corresponding author:** alwyn.williams@outlook.com

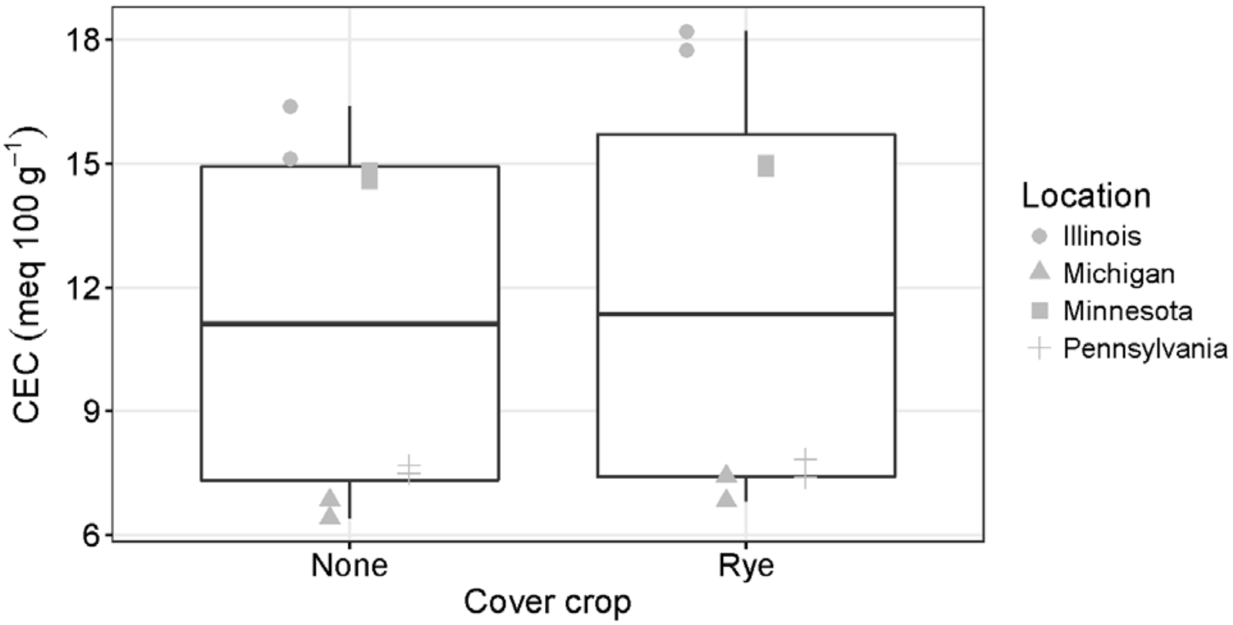

Figure S1. Soil cation exchange capacity (CEC, 0-10 cm depth) in 2015 by cover crop treatment.

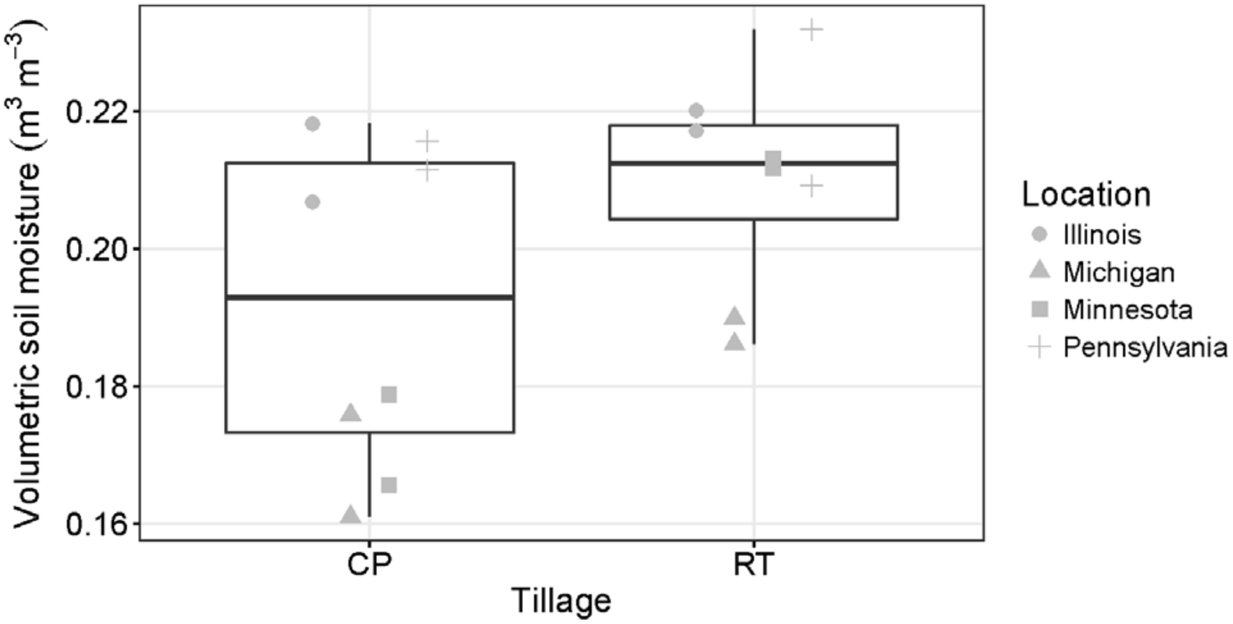

Figure S2. Mean daily soil moisture (0-10 cm depth) between the maize six leaf stage (V6) and tasseling (VT) under chisel plow (CP) and ridge tillage (RT) over 2012-2015.

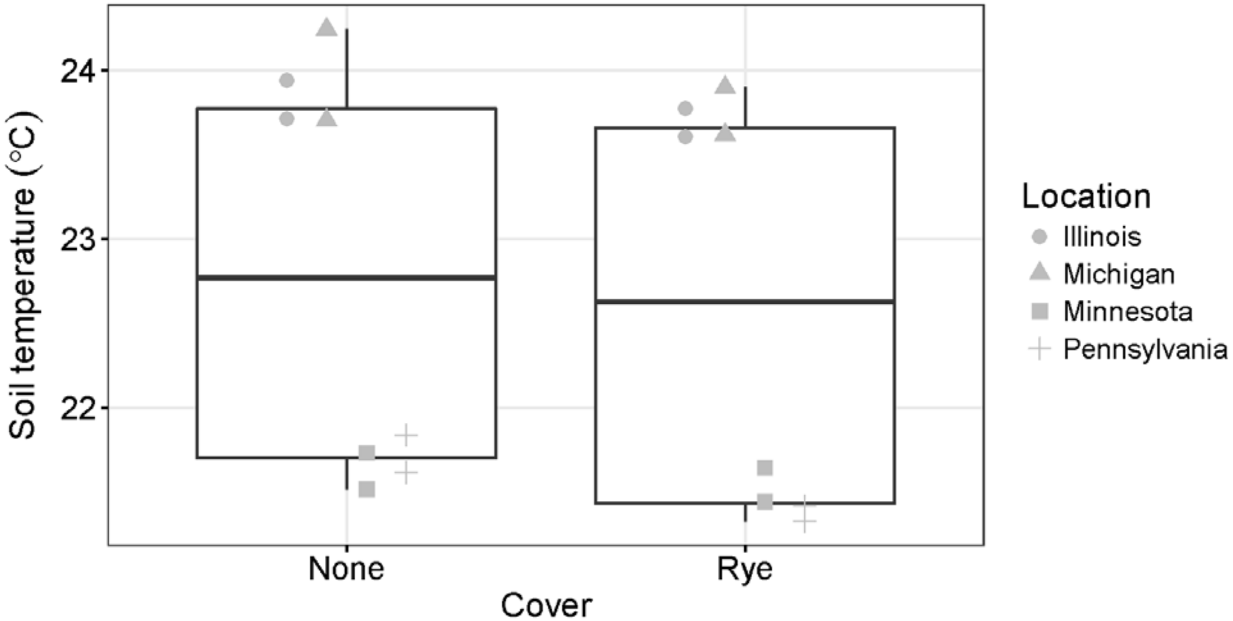

Figure S3. Mean daily soil temperature (0-10 cm depth) between the maize six leaf stage (V6) and tasseling (VT) by cover crop treatment over 2012-2015.

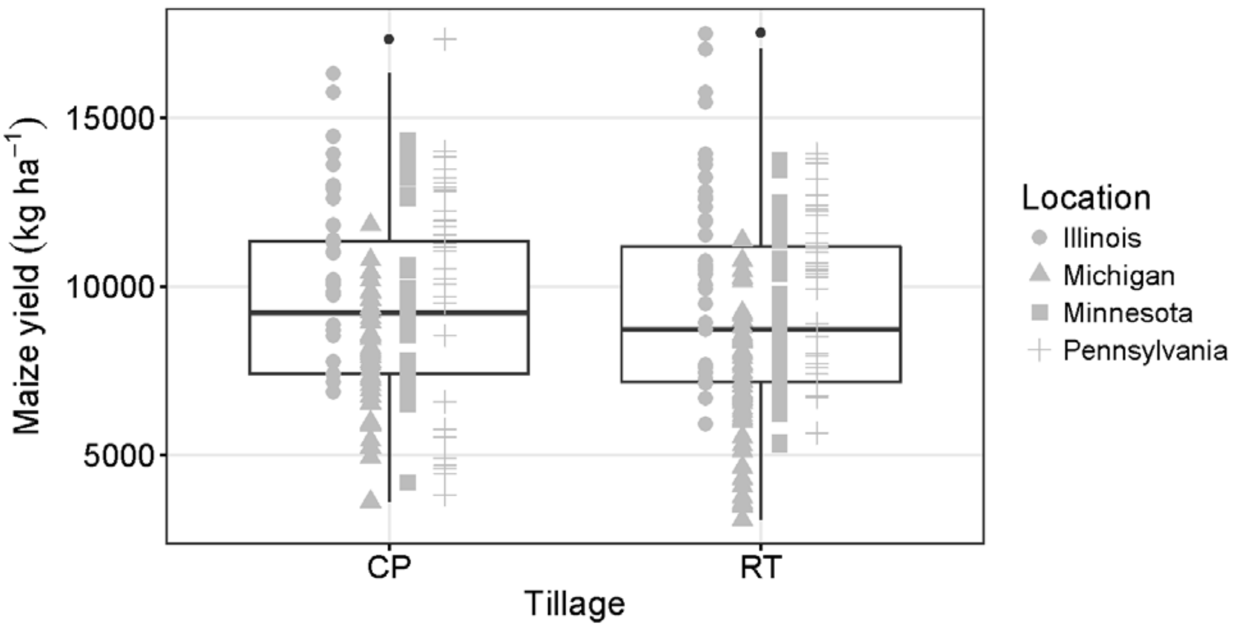

Figure S4. Maize yields under chisel plow (CP) and ridge tillage (RT) over 2012-2015.

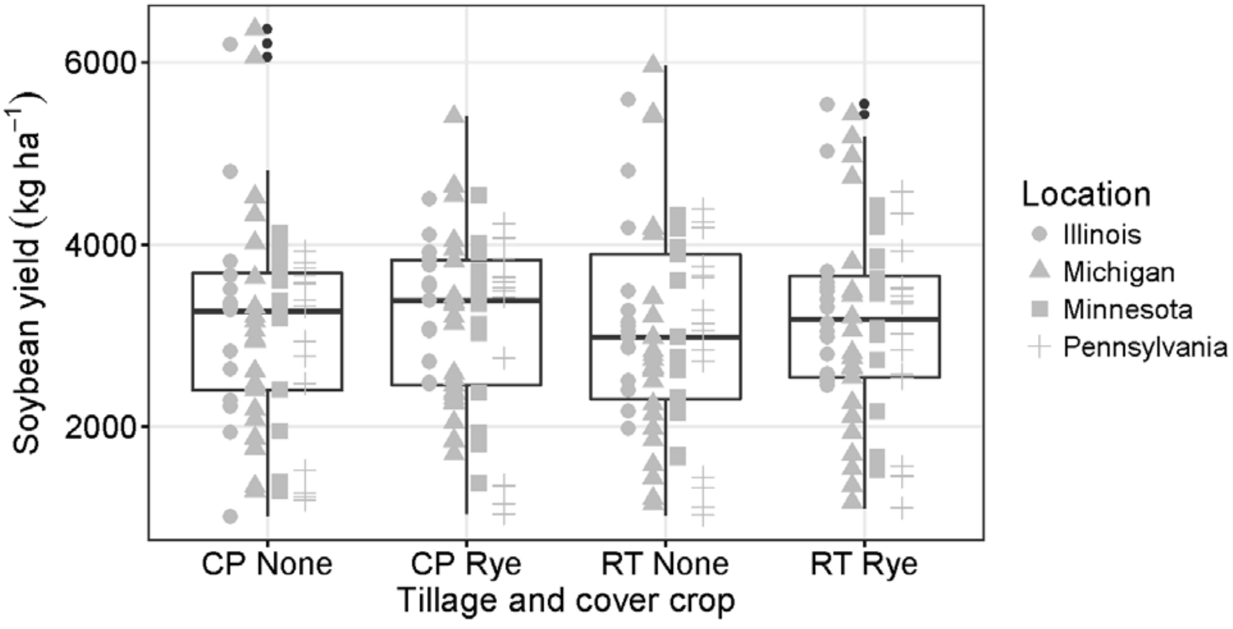

Figure S5. Soybean yields by tillage and cover crop treatments over 2012-2015. CP = chisel plow; RT = ridge tillage.
